# Electrospun Polymer Materials with Fungicidal Activity: A Review

**DOI:** 10.3390/molecules27175738

**Published:** 2022-09-05

**Authors:** Nasko Nachev, Mariya Spasova, Nevena Manolova, Iliya Rashkov, Mladen Naydenov

**Affiliations:** 1 Laboratory of Bioactive Polymers (LBAP), Institute of Polymers, Bulgarian Academy of Sciences, Acad. G. Bonchev St., bl. 103A, BG-1113 Sofia, Bulgaria; 2 Department of Microbiology, Agricultural University, BG-4000 Plovdiv, Bulgaria

**Keywords:** electrospinning, biopolymers, *Phaeomoniella chlamydospora*, *Phaeoacremonium aleophilum*, esca, green agriculture

## Abstract

In recent years, there has been special interest in innovative technologies such as polymer melt or solution electrospinning, electrospraying, centrifugal electrospinning, coaxial electrospinning, and others. Applying these electrokinetic methods, micro- or nanofibrous materials with high specific surface area, high porosity, and various designs for diverse applications could be created. By using these techniques it is possible to obtain fibrous materials from both synthetic and natural biocompatible and biodegradable polymers, harmless to the environment. Incorporation of low-molecular substances with biological activity (e.g., antimicrobial, antifungal) is easily feasible. Moreover, biocontrol agents, able to suppress the development and growth of plant pathogens, have been embedded in the fibrous materials as well. The application of such nanotechnologies for the creation of plant protection products is an extremely promising new direction. This review emphasizes the recent progress in the development of electrospun fungicidal dressings and their potential to be applied in modern agriculture.

## 1. Introduction

The preparation of micro- and nanofibrous materials is an area that has been the subject of increasing scientific interest in recent years. This is due to the fact that these materials are made up of fibers with diameters in the nano- and microscale and possess both a large specific surface area and a small pore size, and are therefore widely used in medicine, pharmacy, cosmetics, electronics, etc. The preparation of micro- and nanofibers can be achieved by using the methods drawing [1,2], template method [3,4,5], phase separation [6], self-assembly [7,8], extrusion [9], electrospinning [10,11,12], and others. Among them, electrospinning is a simple, cost-effective, flexible, and effective method to obtain micro- and nanofibers with desired composition and morphology. An advantage of this method is that a wide variety of functional components of organic or inorganic origin can be incorporated in the fibers [13]. In the present review, the development and the progress in the electrospinning process are presented, and the potential applications of the electrospun fibrous materials in agriculture and, in particular, their potential to protect vineyards against *P. chlamydospora* and *P. aleophilum* are summarized and discussed.

## 2. Crop Diseases Caused by Fungi

Nowadays, plant diseases are a worldwide problem. A disease is any disturbance in the growth and development of a plant caused by unfavorable soil and climatic conditions, by fungi, bacteria, viruses, mycoplasmas, etc. Cultivated plants are essential for humans to satisfy various needs. They can be grown both to obtain food and for the needs of pharmacy, light industry, etc.

Diseases of cultivated plants, as well as the adverse influence of various pests, cause damage to agricultural crops every year. In most cases, they are specialized to certain crops or a group of crops, and if precautions are not taken to combat them, they can cause a severe decrease in yields or fail the crop completely.

Plant diseases are mainly caused by various plant pathogens: bacteria, viruses, fungi. Fungi cause more plant diseases than any other group of pests. Controlling them is difficult, as they can be easily spread by spores, which are produced in abundance. These spores land on plants through wind, water, soil, birds, and insects. Among the factors that favor the spread of these diseases through fungal spores are warm weather and high relative humidity.

Esca disease is one of the earliest known diseases of grapevines. It was already described by the Romans and the ancient Greeks and is considered the oldest vine disease [14]. It is widespread in all wine-growing countries and occurs worldwide, causing huge economic losses [15]. In the last three decades, the attention to esca has been extremely intensified worldwide—the cases of manifestation of the disease have increased significantly. The disease is spreading rapidly and is a real threat to vineyards in Europe. A drastic increase in diseased esca has been reported in Italy [16,17] and in Germany [18]. A total of 80% of vineyards in Tuscany (Italy) show foliar symptoms of esca [19]. It is known that the causative agents of the disease are the fungi, mostly of the species *P. chlamydospora* and *P. aleophilum* [20,21]. The symptoms of the disease are manifested in an acute or chronic form, which can affect different parts of the same plant [16]. In addition, fruits can be affected as well [22]. The wounds obtained during the pruning of the vines are considered to be the “main entrance” for the penetration of the fungal spores into the plant. The disease was first successfully controlled in 1903 when sodium arsenite was used as an insecticide on grapes. Whole-crop spraying with sodium arsenite during the active season was long considered the only way to control esca disease. Since 2003, sodium arsenite was declared highly toxic and carcinogenic and has been banned for agricultural use in Europe. Thus, in practice, there are no known approaches and means of direct fighting with esca, which from an economic point of view represents a huge problem for the preservation of the vineyard massifs. Currently, only preventive approaches are applied, the most frequently applied being the removal of severely damaged plants and their replacement with new ones. This is an extremely costly approach—it has been estimated that with an annual replacement of about 1% of plants killed by vine wood diseases, the financial costs will exceed EUR 1 billion worldwide [23]. Therefore, in order to prevent losses of grape crops and losses to the wine industry, it is imperative to develop and implement new approaches and products to fight esca.

In the last few decades, polymer materials have been increasingly used in agriculture [24,25]. Smart polymer systems have contributed significantly to the development of the agricultural industry by increasing the effectiveness of pesticides, herbicides, and fertilizers through their controlled release and the use of smaller amounts of them. Superabsorbent polymeric materials are used as soil improvers to control drought, while polycationic polymers are used in bioengineered plant breeding.

An alternative to the chemical products used for plant protection is the use of natural substances and/or microorganisms exhibiting biological activity towards plants and phytopathogenic microorganisms. This activity consists of suppressing or stopping the development of phytopathogens and stimulating the plants. The stimulating effect is expressed in accelerating their growth and increasing their resistance to phytopathogenic microorganisms [26].

## 3. Electrospinning

The first description of the electrospinning process was given in 1902 when J.F. Cooley applied for a patent entitled “Apparatus for electrically dispersing fluids” [27]. In his patent (US 692631), he described a method using high-voltage sources to obtain fibers. The next significant scientific development was achieved by Zeleny [28], who published his work on the behavior of liquid droplets at the end of metallic capillaries. His work marked the beginning of efforts to mathematically model the behavior of liquids under the action of electrostatic forces In the 1930s, Formhals patented his invention for industrial production of artificial filaments plastics [29]. Between 1964 and 1969, Taylor laid the theoretical foundation of the electrospinning process [30]. His publication contributes to the understanding of the process by mathematically modeling the shape of the cone formed by the drop of liquid under the action of electrostatic forces. This characteristic droplet shape is still known as the Taylor cone.

Nowadays, electrospinning has established itself as one of the most promising and effective methods for obtaining continuous micro- and nanofibers [31]. Interest in the method of electrospinning in the period 2000–2021 grew many times over. According to ScienceDirect^®^, the number of scientific publications in the database for the year 2000 when searching for the keyword “electrospinning” is eight, and in 2021 this number increases to 5695.

Depending on the viscosity of the solutions, two processes are distinguished: electrospraying or electrospinning. When using diluted polymer solutions, electrospraying is observed—obtaining micro- or nano-sized particles. When an electric field is applied to concentrated polymer solutions (solutions with sufficiently high viscosity), a fiber formation process is observed. This process is known as electrospinning. Polymers have flexible chains in solution. They form the so-called “random coils”. In highly concentrated solutions of such polymers, the statistical globules are interpenetrating and form a physical network of intertwined segments of the polymer chains. This entanglement is essential for forming an elastic network and forming a micro- or nanoscale fiber (diameters in the micro- or nanoscale and hundreds of meters in length) [32].

In both processes, under the action of electrostatic forces, the surface of the drop is deformed and acquires a conical shape (Taylor’s cone) [30]. When the applied voltage is high enough (inducing enough repulsion of the charges to overcome the surface tension of the polymer solution), a jet of liquid is ejected from the tip of the needle. Initially, the trajectory of the jet is straight and stable, then it undergoes separation processes in electrospraying and stretching and bending to obtain a long and thin filament in electrospinning [33].

Fiber formation by electrospinning is a process that is affected by a number of factors such as spinning solution parameters and electrospinning process parameters. When using a polymer with a high molar mass, the viscosity of the solution increases. In order to observe an electrospinning process which will lead to defect-free fiber formation, it is necessary for the polymer solution to be sufficiently viscous, since at lower viscosity values, fibers with defects are obtained. Shenoy et al. reported that that complete, stable fiber formation occurs at >2.5 entanglements per chain [32].

The viscosity of a solution is closely related to its concentration. Increasing the viscosity or the solution concentration results in a more uniform fiber formation and narrow distribution of the mean fiber diameter [34]. In solutions with low viscosities, the surface tension is the determining factor and then only spheres or fibers with spherical defects are obtained, while above a certain critical concentration, a continuous fibrous structure is obtained and its morphology is affected by the concentration of the solution [35].

The parameters of the electrospinning process, which influence the morphology of the fibers, are the electric voltage, the feeding rate of the spinning solution, the distance between the tip of the capillary and the collector, the collector rotation speed, the type of collector used, etc. The applied voltage is a major factor without which the process cannot be carried out. Typically, a positive or negative voltage of over 6 kV is required for the jet to form a Taylor cone [36]. Uniform defect-free fibers are obtained above a certain critical value of solution feed rates. Increasing the velocity above the critical one leads to the formation of particles [37]. Another key parameter for fiber formation is the distance between the tip of the capillary and the collector. It was found that when this gap is reduced, the time of flight of the jet is shortened. In brief, if the distance is too short, the solvent evaporation time is insufficient and the fibers fail to dry before reaching the collector. In this case, solvent-containing fibers are deposited [38,39]. For this reason, it is necessary to find an optimal gap for the solvent to evaporate and dry fibers to be deposited on the collector. Environmental parameters such as temperature and air humidity also influence fiber morphology and diameter. It was found that with increasing temperature, fibers with smaller diameters are obtained, which is due to a decrease in the viscosity of the polymer solutions at high temperatures [40]. Predominantly, electrospinning of polymer solutions is carried out in air.

The use of different types of collectors and focusing devices can affect the jet trajectory and hence the morphology and orientation of the resulting fibers. The simplest collector used in electrospinning is a stationary metal plate or foil located at a certain distance from the needle. When using this type of collector, the fibers are usually deposited randomly on the metal plate. It has been established that a certain control of the orientation of the resulting mat is achieved with the rotary movement of the collector. Different types of collectors used in electrospinning are solid cylinder, solid cylinder with a wire, a disc, blades, etc. The advantage of using a drum or disc collector is that oriented fibers can be prepared, and long oriented bundles can be obtained. The disadvantages of their use, however, are that the degree of fiber orientation decreases with increasing layer thickness. By using a wire-wrapped or wire-built collector, highly oriented fibers can be obtained. Very good fiber ordering is obtained when blade-type collectors are used [41]. Needle electrodes were also made with the aim of self-organizing the fibers into bundles and yarns. Comb-type electrode with a different number of interchangeable needles, from one to five, has been developed as well. The body of the comb is made of solid wood, and the electrode needles are made of stainless steel [42].

### Applications

In recent years, the interest in the production of micro- and nanofibrous materials by the electrospinning method has been continuously increasing. This is due to the possibility of imparting desired properties to the obtained fibrous materials, such as biodegradability, biocompatibility, mechanical strength, etc. Electrospun fibrous materials are known to possess a large specific surface area (10^3^ m^2^/g) and small pore size and spatial structure [43]. The potential applications of these materials as filter media [44,45], tissue engineering scaffolds [46,47], carriers of active substances [48,49], protective clothing [50], and sensors [51] are the subject of increasing interest.

Polymers are all around us, as polymer products have widely entered our lives. Natural polymers such as cellulose and starch are widely used in the food industry and for the production of paper and packaging. Obtaining polymers from renewable sources changes production and recycling technologies, providing opportunities to create environmentally friendly materials that do not pollute the environment, degrading significantly faster than synthetic polymers. Therefore, research in the field of polymers is necessary not only for the development of such branches of industry as the production of products based on polymers, but they are an important and key component of interdisciplinary research related to the development of high technologies, nanotechnology, biotechnology, medicine and pharmacy, and environmental protection.

Over the past few decades, there has been a continued scientific and industrial interest in biocompatible and biodegradable polymers, especially in saturated polyesters. This is due to the fact that they can be obtained from renewable sources and also to their valuable physicomechanical properties. One of the most attractive synthetic polymers are the aliphatic polyesters such as poly(lactic acid), poly(glycolic acid), and their copolymers. Aliphatic polyesters are characterized by one main advantage—they degrade in the human body and in the environment through the hydrolysis of their ester bond. In addition, they are compatible with human organs and tissues. That is why these synthetic polymers are considered to be one of the most promising for obtaining polymer products for the needs of medicine and pharmacy [52,53]. These polymers are used to produce absorbable sutures, controlled drug release systems, and orthopedic fixation devices (implants) such as pins, screws, and plates [54,55]. Their wide application to make suitable polymeric products for the needs of tissue engineering is known as well [56].

Fibrous materials obtained by applying the method of electrospinning/electrospraying solutions of aliphatic polyesters are characterized by a set of desirable properties such as biocompatibility, degradability, similarity to the extracellular matrix of natural tissues, and excellent physicomechanical properties. In the literature, there are quite a few studies related to the influence of the various parameters of the electrospinning process on the diameters and morphology of the obtained polylactic acid (PLA) fibers. Micro- and nanofibrous materials based on homo- and copolymers of PLA are promising candidates for application in cell and tissue engineering [57,58] to create new drug carriers [59], for the restoration of bone tissue [60], for water purification [61], etc.

In the last ten years, there has been a growing interest in PLA electrospinning, as evidenced by the growing number of scientific publications. The number of scientific publications on PLA electrospinning according to ScienceDirect data in 2011 was 143, reaching nearly 1000 in 2021. In 2007, Spasova and co-authors successfully electrospun poly(L-Lactic acid) (PLLA) and PLLA/polyethylene glycol (PEG) mixed solutions [62]. Increasing the amount of PEG in the solution resulted in thinner and more hydrophilic fibers with average diameters of 330 nm. The thinnest fibers were obtained when using a PLLA/PEG spinning solution with a concentration of 5 wt% and a PLLA to PEG ratio of 70/30. The process takes place at a constant value of the applied voltage of 11 kV. Electrospinning under these conditions, however, leads to the appearance of defects along the fiber length. Two types of cells—fibroblasts and osteoblasts—were used to conduct biological tests to establish the cellular compatibility of the obtained materials. It was found that the osteoblasts began to organize into tissue-like structures, especially in the materials with a higher content of the water-soluble polymer.

By using electrospinning, the valuable properties of polylactic acid and its copolymers are combined with natural polymers such as chitosan, which has its own biological activity [63,64]. PLLA/PEG fibers were obtained which were coated with chitosan [63]. The presence of chitosan coating was demonstrated by SEM and fluorescence microscopy. The behavior of the obtained hybrid fibrous materials when placed in contact with human blood was investigated. Chitosan-coated materials have been found to induce blood clotting. The microbiological tests carried out against *Staphylococcus aureus* show that the chitosan coating imparts antibacterial activity to the obtained mats. Combining hemostatic with antibacterial properties makes these materials suitable for treating wound surfaces.

Toncheva and co-authors created PLLA and PLLA/PEG membranes containing one or more drug substances, such as diclofenac sodium, lidocaine hydrochloride, benzalkonium chloride, or combinations thereof. The incorporation of low-molecular-weight salts contributes to the improvement of the conductivity of the spinning solutions and the preparation of oriented fibers with antimicrobial properties [42,65]. In addition, Ignatova et al. found that fibers based on PLA and 8-hydroxyquinoline-2-carboxaldehyde possess antitumor activity against HeLa cancer cells [66].

There are two main approaches to electrospinning drug-loaded materials. In one approach, a mixed spinning solution containing the polymers and drugs is prepared [42,65]. In the other approach, electrospinning is performed from two polymer solutions loaded into two syringes, with a separate drug incorporated into each solution [67,68]. The advantages of the electrospinning method when using two syringes containing solutions of different biologically active substances is that they do not ionically interact with each other. The obtained new materials can find applications in biomedicine and pharmacy.

Electrospraying is a technique that can be used to produce micro- and nanoparticles. Virsovska and co-authors first compared the application of the electrospinning technique with the simultaneous electrospinning/electrospraying technique on PLLA and nanosized ZnO mats [69]. To obtain the “in” type materials, ZnO was added to a PLLA solution and the resulting suspension was subjected to electrospinning. The “on” type materials were obtained by simultaneous electrospinning and electrospraying, using two separate pumps to deliver the two solutions. One syringe contains a concentrated PLLA solution and the other a suspension of ZnO in a dilute PLLA solution. It was found that combining the two methods leads to the preparation of hybrid fibrous materials with improved photocatalytic and antibacterial activity. New self-cleaning hybrid fibrous materials based on PLLA decorated with nanosized ZnO and expanded graphite or fullerene (C60) were also obtained [70].

By using the electrospinning method, biologically active substances of natural origin can easily be incorporated into fibrous materials. Curcumin is a polyphenolic compound extracted from the roots of the turmeric plant (*Curcuma longa* L., family Zingiberaceae). It has antitumor, antioxidant, anti-inflammatory, and antimicrobial properties [71,72]. Yakub and co-authors successfully incorporated curcumin into fibers of PLLA and PVP or PEG [73,74]. Curcumin incorporated into the fibers is protected from photodegradation. The presence of polymers that can form hydrogen bonds with curcumin facilitates its extraction. The obtained new materials, based on PLLA, possess both good mechanical properties and antibacterial, antioxidant, and anticoagulant activities.

By exploiting the property of PLA to form stereocomplexes, novel fibrous materials of PLLA and PDLA [75,76] and PLLA and PDLA-b-PBS [77,78] have been obtained, which have an increased melting temperature and therefore are suitable for biomedical applications. Moreover, PLA fibrous materials were prepared, on which a polyelectrolyte complex of *N*-carboxyethyl chitosan and polyoxyethylene-*b*-quaternized poly-2-(dimethylamino)ethyl methacrylate was formed, which possesses hemostatic properties and reduces the adhesion of pathogenic microorganisms [79].

Incorporation of metal oxide nanoparticles into fibers by electrospinning is a promising method for preserving their properties and imparting specific properties to hybrid fibrous materials such as antibacterial, antifungal, antitumor, antioxidant, anti-inflammatory, self-cleaning, and other activities [80,81].

## 4. Electrospun Fibrous Materials with Antifungal Activity against Esca Disease

Modern agriculture requires not only the creation of new means of plant protection with low toxicity to non-pathogenic soil microorganisms, but also the search for rational solutions and approaches to improve the existing ones. Modern plant protection products must be designed in such a way as to achieve the desired biological effect without unwanted side effects and impacts. The application of nanotechnology in the field of agriculture is growing gradually. Obtaining nanofibers by electrospinning and applying them for agricultural purposes has many advantages due to the fact that these fibers are relatively easy to obtain, possess pores, have a large specific surface area, and diverse bioagents can be effectively incorporated into them [82]. Therefore, they are used for the protection of seeds, plants, and soils, as well as in the detection of harmful compounds [25,83]. In addition, biosensors [84,85], protective clothing for agricultural workers [86], packaging [87,88], etc., can be made on their basis.

In the literature, for the first time, in 2015, the creation of biodegradable and biocompatible nano-textured membranes using electrospun soy protein/polyvinyl alcohol and soy protein/polycaprolactone nanofibers for prevention of esca fungi invasion into pruning cuts and wounds of vines was reported [89]. The electrospinning time was varied in order to control the pore size and thereby a protective layer against fungi penetration was formed. The created novel materials are radically different from those known up to date and could be used to protect plants against fungal attack through pruning locations and help to prevent diseases in grapevines. The prepared adhesive membranes can withstand strong wind without being blown off while allowing sufficient porosity for plants to breathe.

As a continuation and development of the work of Sett and co-authors, another research group obtained poly(lactide-*co*-glycolide) (PLGA) and poly(butadiene adipate-*co*-terephthalate) PBAT electrospun materials with polyhexamethyleneguanidine (PHMG) incorporated to inhibit the penetration of *P. chlamydospora* spores [90]. The used concept consists of blocking of fungal spores to penetrate trough the pruning wounds of vine plants while allowing the exchange of air and moisture trough the nonwovens. Therefore, the authors selected the electrospinning for the creation of the blocking and breathable membranes. Moreover, they choose the work with compostable polymer which could remain in vineyards after use and degrades in soil with time. For this purpose, typical compostable polymers such as PLGA and PBAT were selected. The electrospinning of the PLGA solution resulted in preparation of fibers without any beads. Increasing the electrospinning time resulted in preparation of nonwoven with increased thickness. All prepared fibrous mats showed almost the same pore size distribution with an average value for the mean flow pore size of about 3.5 ± 0.5 μm. The barrier efficiency of the nonwovens against fungal spores was studied in model experiments with aerosol particles ranging in sizes from 2 to 0.25 μm. It was found that the PLGA membranes with a thickness of 86 μm and more possessed barrier efficiency of 100% for all aerosol particles analyzed. Quantitative barrier efficiency was also investigated for electrospun fibrous materials of PBAT and PBAT/PHMG as well. The authors conclude that by the results obtained from the model investigations, all the obtained nonwovens should be ideal barrier materials against *P. chlamydospora* spores of 1–1.5 μm width and 3–5 μm length. However, the results from barrier efficiencies with living fungal spores showed that PLGA and PBAT fibrous mats supported and developed colonies of *P. chlamydospora* on the surface and the spores also penetrated the nonwoven while the amount of the penetrated spores depend upon the thickness of the nonwovens. A 100% barrier efficiency was not achieved in any experiment with living *P. chlamydospora* spores. However, the incorporation of PHMG into PBAT fibers resulted in creation of nonwovens with antifungal properties and blocked the penetration of esca spores to the substrate.

Another approach to create novel materials with strong antifungal activity to protect vines from penetration and infection of different fungi relies on the incorporation of 8-hydroxyquinoline derivatives into bio-based fibrous materials prepared by electrospinning. This way, the valuable biological properties of 8-hydroxyquinolines with the advantages of electrospun materials can be combined. It is well known that 8-hydroxyquinoline and its derivatives manifest antibacterial, antifungal, and antiviral activities [91,92,93] and are of low toxicity to humans [94]. They have great potential in the development of new therapeutics for the treatment of bacterial and fungal diseases resistant to traditional chemotherapeutics, as well as for the treatment of some neurodegenerative diseases [95].

Recently, we showed that the combination of water-soluble polymers and 8-hydroxyquinoline derivatives results in obtaining of stable solutions with antifungal activity suitable for applications in agriculture. The minimum inhibitory concentration (MIC) of 5-Cl8Q against *P. chlamydospora* and *P. aleophilum* was found to be 0.75 μg/ml for both strains [96].

**Figure 1:**
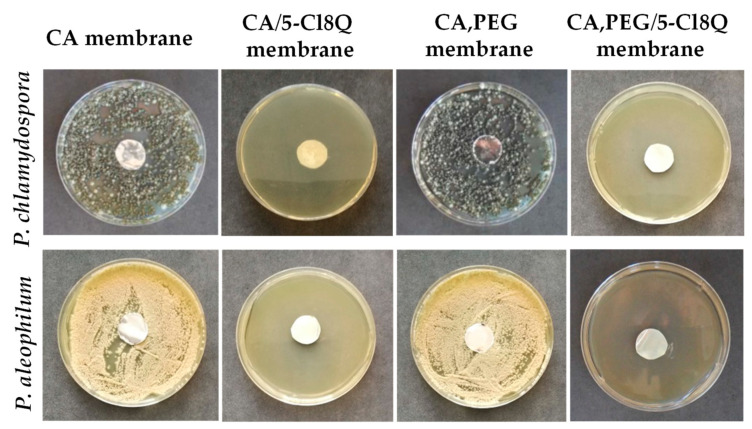
Digital images of the zones of inhibition against *P. chlamydospora* and *P. aleophilum* after contact of the membranes with fungi cells. The membrane type is indicated at the top of each column. The cell type is marked in the left of each row [97].

Novel micro- and nanofibrous membranes from cellulose acetate (CA) and cellulose acetate/polyethylene glycol (CA/PEG) containing 5-chloro-8-hydroxyquinolinol (5-Cl8Q) were successfully prepared by electrospinning [97]. SEM analysis was used to investigate the morphology of the obtained membranes. The CA fibers were defect-free while the incorporation of PEG in CA resulted in decrease of the diameters of the obtained fibers which is most probably due to the decrease in the solution viscosity by adding a lower-molecular-weight polymer to the spinning solution. The incorporation the 5-Cl8Q resulted in insignificant decrease of the fiber diameters. The presence of the biologically active substance was proved by FTIR. The presence of a new band characteristic for the quinoline ring was detected in the spectra of CA/5-Cl8Q and CA,PEG/5-Cl8Q membranes. Moreover, it was determined that the addition of PEG resulted in hydrophilization and decrease of the water contact angle to 0°; however, it resulted in significant decrease of the mechanical properties of the obtained membranes. The antifungal activity of the prepared fibrous membranes was tested against *P. chlamydospora* and *P. aleophilum*. The results obtained by determination of the zones of inhibition after contact of the fibrous materials with the fungal cells are shown in Figure 1.

The neat CA and CA,PEG fibers did not affected the fungal growth and did not exhibit any antifungal activity. Contrariwise, the addition of 5-Cl8Q in the fibrous mats placed in contact with *P. chlamydospora* and *P. aleophilum* resulted in complete inhibition for all fungi. The detected wide zones of inhibition around all fibrous mats containing 5-Cl8Q are evidence that the incorporated bioactive compound imparts a considerable antifungal effect against *P. chlamydospora* and *P. aleophilum* fungi to the prepared novel fibrous membranes.

As a continuation and improvement of the obtained fibrous materials on the basis of CA and CA,PEG a biopolymer with better mechanical properties for the creation of the fibrous membranes was selected. A synthetic biodegradable polymer, PLA, was chosen because it possesses good mechanical properties and is highly biocompatible and biodegradable. Two types of 8-hydroxyquinoline derivatives were used—5-chloro-8-hydroxyquinoline and potassium 5-nitro-8-hydroxyquinoline. They were selected because of their low MICs against *P. chlamydospora* and *P. aleophilum* fungi [98].

**Figure 2:**
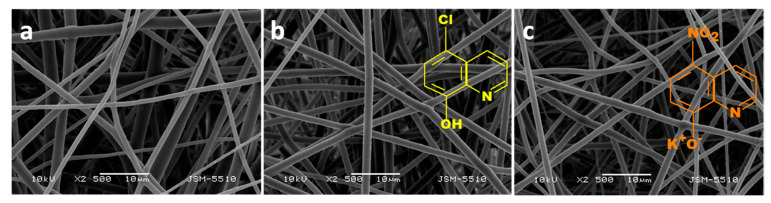
Representative SEM images of fibers of electrospun fibrous materials of (**a**) PLLA, (**b**) PLLA/5-Cl8Q, and (**c**) PLLA/K5N8Q; magnification ×2500 [98].

The fabricated PLLA, PLLA/5-Cl8Q, and PLLA/K5N8Q fibers are shown in Figure 2. The obtained PLA fibers were cylindrical and defect-free with mean fiber diameters of 1045 ± 320 nm. The incorporation of the 8-hydroxyquinoline derivatives does not lead to significant change in morphology and mean fiber diameter of the obtained composite fibers. The performed XRD analysis proved that PLLA/5-Cl8Q and PLLA/K5N8Q composite fibrous materials are amorphous while the initial 5-Cl8Q and K5N8Q (powder) are highly crystalline. This finding shows that the electrospinning process could result in amorphization of some highly crystalline substances.

The mechanical properties of the PLLA/5-Cl8Q and PLLA/K5N8Q composite fibers manifest characteristics similar to the PLLA fibrous mats with tensile strength ~2.5 MPa.

The antifungal activity of the obtained electrospun membranes was determined by performing antifungal tests against *P. chlamydospora* and *P. aleophilum*. Complete inhibition of the *P. chlamydospora* spores was observed in the case of PLLA/5-Cl8Q. Wide inhibition zone of ~4.7 cm around the PLLA/5-Cl8Q disc placed in contact with *P. aleophilum* was determined. Around the discs incorporated with K5N8Q, the inhibition zones of 6.2 cm and 4.0 cm against *P. chlamydospora* and *P. aleophilum* were measured.

The barrier efficacy of obtained fibrous materials was determined. From each mat, a disc with diameter 45 mm and thickness ca. 1 μm was cut. Then, a 20 mL conidia suspension was passed through each fibrous disc by using a filtration. The initial spore concentration was 1 × 10^7^ spores/mL, and after passing through the mats the spore concentration decreased by four times. After performing the filtration experiments, the used mats were placed in a Petri dish in order to determine the growth of the remaining in the fibrous discs’ fungi. Compete fungal inhibition was observed in electrospun composite materials containing the 8-hydroxyquinoline derivatives.

**Figure 3:**
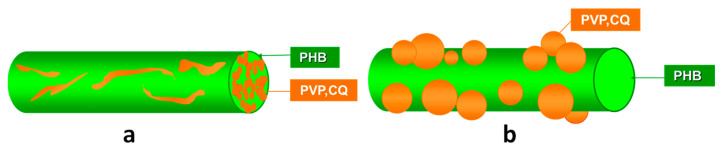
Schematic representation of fibers: (**a**) PVP*in*PHB fiber containing CQ in the bulk (PVP,CQ*in*PHB) and (**b**) PHB fiber decorated with PVP,CQ particles on the surface (PVP,CQ*on*PHB) [99].

The potential of 8-hydroxyquinoline derivatives to serve as antifungal substances for plant protection was further explored with 5-chloro-7-iodo-8-hydroxyquinoline (Clioquinol, CQ) [99]. Until this first report, no data in the literature on the antifungal activity of CQ against *P. chlamydospora* and *P. aleophilum* were reported. Initially, the MICs values of CQ were determined. They were 10 and 1 μg/mL for the both fungi. After that, CQ was added to the spinning solutions of poly(3-hydroxybutyrate) (PHB)and/or polyvinylpyrrolidone. Two types of materials were obtained by using one-pot electrospinning (“*in*” strategy) or electrospinning in conjunction with electrospraying (“*on*” strategy). A schematic representation of the prepared fibrous materials from PHB and PVP containing CQ of different designs is shown in Figure 3.

The electrospinning of solutions of PHB and/or PVP with/without CQ resulted in preparation of cylindrical and defect-free fibers. The mean fiber diameters of the PHB,PVP*in*PHB and PVP,CQ*in*PHB fibers were 760 ± 200 nm, 480 ± 110 nm, and 470 ± 110 nm, respectively. When PHB fibers were decorated with PVP,CQ particles, it was observed that the particles had spherical shape with mean particle size of 490 ± 150 nm. Thus obtained materials were further analyzed by X-ray photoelectron spectroscopy and attenuated total reflection Fourier- transform infrared spectroscopy. It was determined that CQ incorporated in the bulk of the fibers or in PVP particles deposited on the fibers was in the amorphous state, which was confirmed by differential scanning calorimetry and X-ray diffraction analysis. The studied in vitro CQ release from the different types of fibers confirmed that CQ was released faster and in a greater amount when incorporated into PVP particles that are deposited on the PHB fibers’ surface than when incorporated in the PVP*in*PHB fibers. The amount of CQ released from the PVP,CQ*in*PHB mats was ca. 83.5%, and for PVP,CQ*on*PHB fibrous materials, the amount of CQ released was 96% for the same time period of 2880 min.

The antifungal activity of the created fibrous materials against the fungi *P. chlamydospora* and *P. aleophilum* was studied. As expected, PHB and PVP*in*PHB mats did not show any significant antifungal activity. The fibrous materials containing CQ, however, exhibited strong antifungal activity against these fungi, and well-defined zones of inhibition of fungal cell growth were detected. All these results manifest that CQ-containing fibrous materials (both “in” and “on” types) exhibited considerable antifungal activity against *P. chlamydospora* and *P. aleophilum* and are promising candidates as active dressings for protection of grapevine against the penetration and growth of the two main causative fungal agents of esca disease.

Different metal nanoparticles have been synthesized in order to be used to control phytopathogenic fungi in agriculture. These metal nanoparticles are considered as good alternatives to the use of chemicals, pesticides, and fungicides that are responsible for environmental pollution and health hazards. Moreover, the use of agrochemical agents may lead to development of resistant pathogens.

Among the metal nanoparticles, ZnO and TiO_2_ possess great potential to fight against diverse type of microorganisms. Therefore, nanoparticles of metal oxides (ZnO or TiO_2_) capable of generating highly reactive radicals can be incorporated into polymer matrices in order to give them biological, electrical, optical, and/or photocatalytic activity, as well as to improve their thermal and physicomechanical properties [100,101]. TiO_2_ is known in the literature to exhibit antibacterial properties against both bacteria and fungi. This is due to its ability when exposed to light in the presence of water vapor to form the highly reactive hydroxyl radicals [HO˙] and superoxide [O_2_^−1^] [102,103]. In addition, ZnO nanoparticles also possess antimicrobial activity against a wide range of bacteria, although the mechanism of their action is not fully understood. They are thought to generate hydrogen peroxide (H_2_O_2_), which is responsible for their antibacterial properties [104,105]. Incorporation of metal oxide nanoparticles into fibers by electrospinning is a promising method for preserving their properties and imparting specific properties to hybrid fiber materials such as antibacterial, antifungal, antitumor, antioxidant, anti-inflammatory, self-cleaning, and other activities [106,107].

**Figure 4:**
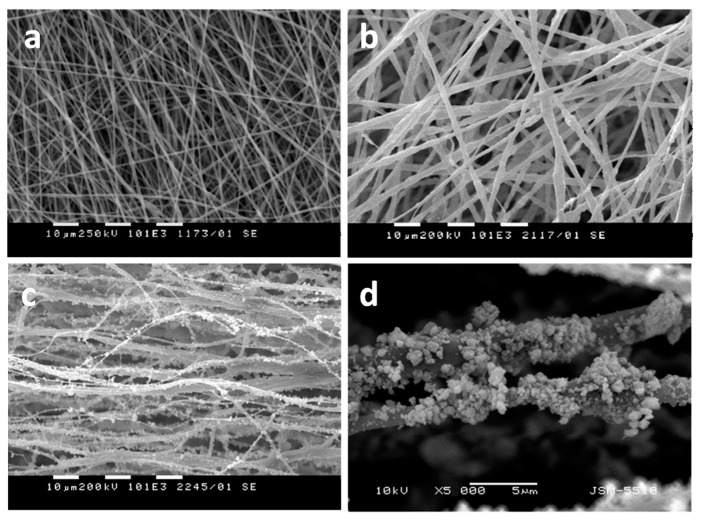
SEM micrographs of fibrous materials: (**a**) PHB (×1000), (**b**) TiO_2_-*in*-PHB (×1000), (**c**) TiO_2_-*on*-PHB (×1000), and (**d**) TiO_2_-*on*-PHB (×5000) [108].

Fibrous materials based on PHB, nanosized TiO_2_—anatase and chitosan oligomers—have also been successfully obtained by simultaneous electrospinning and electrospraying [108]. One-pot electrospinning of a dispersion of nanosized TiO_2_ nanoparticles in PHB solution resulted in obtaining of materials with design type “in”, in which TiO_2_ was incorporated in the fibers. Simultaneous electrospinning of solution of PHB and electrospraying of dispersion of nanosized TiO_2_ and COS resulted in the fabrication of materials with design type “on”, consisting of PHB fibers on which TiO_2_ was deposited on the fibers’ surface. The morphology of the obtained fibers is shown in Figure 4. It was determined that the TiO_2_ particles deposited onto the PHB fibers possessed spherical form with small (ca. 20 nm) and large (500 nm) particle diameters (Figure 4c,d).

**Figure 5:**
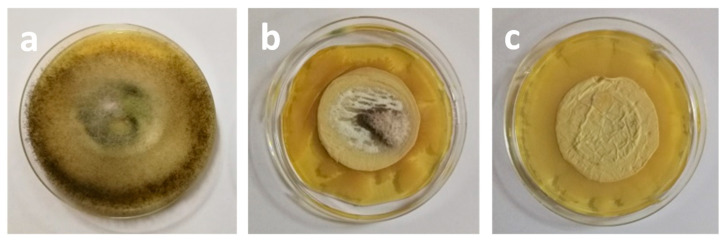
Digital images of the growth of *P. chlamydospora* on the fibrous materials: (**a**) PHB, (**b**) TiO_2_-*in*-PHB, and (**c**) TiO_2_-*on*-PHB [108].

The barrier efficacy of PHB, TiO_2_-*in*-PHB, and TiO_2_-*on*-PHB fibrous materials against the penetration of *P. chlamydospora* conidia was studied. A 20 mL conidia suspension with concentration of 1 × 10^7^ conidia/mL was passed through each fibrous material by using a filtration device. After passing through the fibrous mats, the conidia concentration decreased and it was 5.7 × 10^4^, 3.3 × 10^3^, and 1.4 × 10^3^ for the PHB, TiO_2_-*in*-PHB, and TiO_2_-*on*-PHB materials, respectively. Then, the used mats in filtration experiments were taken off and were placed on a surface of a solid agar in a Petri dish for 96 h at 28 °C under light irradiation, and then the fungal growth was determined. The results of the antifungal activity are shown in Figure 5. It was observed that the PHB mat developed *P. chlamydospora* colonies and the entire surface of the Petri dish was occupied by fungi (Figure 5a). As seen from Figure 5b, the TiO_2_-*in*-PHB mat possessed antifungal activity. However, some fungal growth was observed. It was assessed that only the TiO_2_-*on*-PHB fibrous mat manifested complete inhibition of growth of the fungi remaining in the fibrous material after the filtration (Figure 5c). Thus, the obtained hybrid materials are promising for application in agriculture as innovative materials for plant protection against penetration and growth of main causative fungi causing esca disease.

Another paper showed the perspective to use ZnO nanoparticles as antifungal agents for preparation of hybrid materials by electrospinning for protection against fungal strains. Electrospun cellulose acetate materials decorated with ZnO nanoparticles with improved water-repellent and antifungal properties were created [109]. Initially, suitable conditions for the fabrication of nano- and microstructured materials from cellulose acetate and cellulose acetate/ZnO from solutions/suspensions by electrospinning/electrospraying were found. The obtained materials were characterized by different methods such as scanning electron microscopy, X-ray diffraction analysis, and contact angle measurements. The antifungal activity of the obtained materials against *Phaeomoniella chlamydospora* was studied as well. It was found that electrospinning of CA solutions with concentration of 10 wt% reproducibly resulted in preparation of defect-free fibers with mean fiber diameter of ~780 nm. The incorporation of ZnO nanoparticles resulted in fabrication of hybrid materials with superhydrophobic properties and with water contact angle value of 152°. It was proved that the materials decorated with ZnO nanoparticles manifest antifungal activity against *P. chlamydospora*. The diameter of the zone of inhibition around the ZnO-*on*-CA mat was 16 mm. This result showed that the decoration of electrospun mats with ZnO particles could lead to preparation of materials with antifungal properties which could be suitable candidates to find potential applications in agriculture for plant protection.

The application of synthetic pesticides in agriculture resulted in long-term negative effects for animals, humans, and the environment. For that reason, the modern agriculture demands the creation of effective and ecologically safe devices in order to diminish the use of pesticides. Being environmentally friendly, beneficial microorganisms possess a great potential to fight against phytopathogens and thus can be used as biocontrol agents instead of harmful chemical compounds. The widely studied fungal species, such as *Penicillum*, *Trichoderma*, *Bacillus, Streptomyces,* could degrade natural polymers such as chitin and chitosan, which are construction elements of the cell walls of certain plant pathogenic fungi [110,111,112,113].

**Figure 6:**
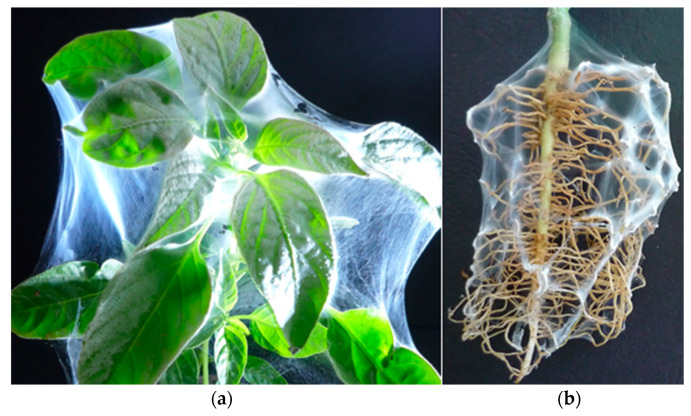
Biohybrid fibers directly electrospun on capsicum leaves and root system.

The successful incorporation of *T. viride* spores into electrospun chitosan/polyethylene oxide and chitosan/polyacrylamide fibers for the creation of biohybrid materials for plant biocontrol was achieved [114]. It was shown that the electrospun biohybrid materials inhibited the growth of diverse phytopathogenic strains (*Fusarium, Alternaria*) when placed in suitable growth conditions. Coating the leaves of plant sprouts (Figure 6a) and plant roots (Figure 6b) with the biohybrids by direct electrospinning was easily performed as well. However, the mechanical properties of the obtained fibrous mats should be improved. Therefore, a novel strategy for preparation of hybrid biomaterials based on an electrospun PLLA mat that possessed good mechanical properties and a bioagent for vineyard protection against pathogens causing esca disease was developed. As bioagent, a beneficial microorganism *T. asperellum* was used. Chitosan with different molecular weight was used for the formation of a film based on chitosan/*T. asperellum* on the PLLA fibers [115].

**Figure 7:**
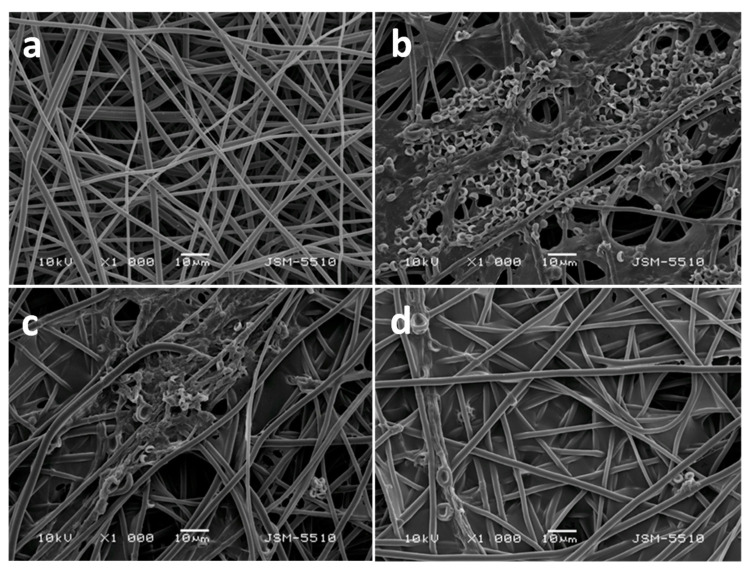
Representative SEM images of (**a**) PLLA mat, (**b**) PLLA mat coated with oligochitosan/*T. asperellum* spores, (**c**) PLLA mat coated with LMW chitosan/*T. asperellum* spores, and (**d**) PLLA mat coated with HMW chitosan/*T. asperellum* spores; magnification ×1000 [115].

The morphology of the obtained PLLA and PLLA mat coated with film of chitosan with different molecular weight (oligochitosan, low-molecular-weight chitosan (LMW) and high-molecular-weight chitosan (HMW)) and *T.* *asperellum* spores are presented in Figure 7. The diameters of the PLLA fibers were 1260 ± 192 nm. Formation of a chitosan coating onto the fibers resulted in increasing of the PLLA fiber diameters coated with HMW chitosan to 1388 nm ± 220 nm. It was observed that the highest number of spores and the most uniform spores’ distribution on the PLLA fibers was achieved when the oligochitosan was used. This is most probably due to the low viscosity of the oligochitosan solution—35 cP—and the ability of the solution to penetrate more easily into the fibrous mat.

Physicomechanical properties of the obtained fibrous mats were determined. The coating with chitosan resulted in improvement of the mechanical properties of the PLLA fibers. The highest value of the tensile strength was measured for the PLLA mat coated with HMW chitosan (6.1 MPa).

**Figure 8:**
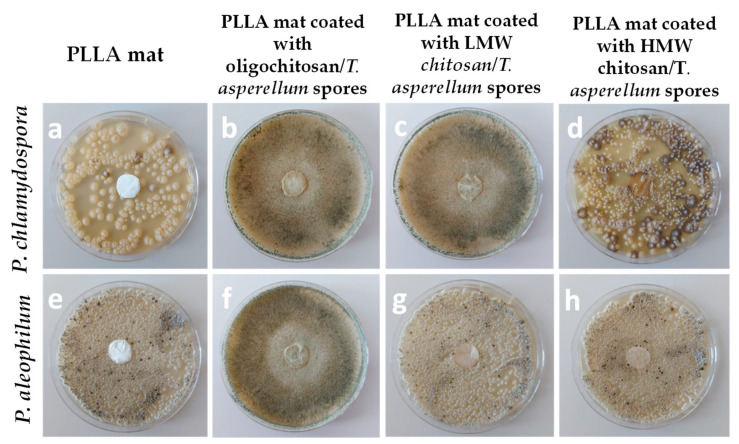
Digital images of the test on the behavior of the mats: PLLA mat, PLLA mat coated with oligochitosan/*T. asperellum* spores, PLLA mat coated with LMW chitosan/*T. asperellum* spores, and PLLA mat coated with HMW chitosan/*T. asperellum* spores against *P. chlamydospora* (**a**–**d**) and *P. aleophilum* (**e**–**h**) [115].

The ability of the biohybrid materials to inhibit the growth of the two pathogenic fungi was assessed. For that reason, from each fibrous mat, discs were cut and placed in contact with *P. chlamydospora* and *P. aleophilum*. The obtained results are shown in Figure 8.

The most effective biomaterial in inhibiting the growth of two pathogenic fungi was the PLLA mat coated with oligochitosan/*T. asperellum* spores. As easily seen, *Trichoderma* mycelium occupied the whole surface of the Petri dish, suppressing the growth of the pathogenic fungi. The PLLA mat coated with LMW chitosan/*T. viride* spores was less effective in inhibiting the growth of *P. chlamydospora* and *P. aleophilum*. It was observed that *Trichoderma* parasitized only on *P. aleophilum*. PLLA mat coated with HMW chitosan/*T. viride* spores did not manifest any suppression of the fungal growth. This is most probably due to the insufficient number of spores incorporated in this material, which was confirmed by the SEM analysis as well. The obtained results show the potential to combine biopolymers with the biocontrol agent by using the electrospinning and film formation techniques for the creation of eco-friendly nanomaterials with good mechanical properties, ensuring the viability of the *T. asperellum* spores which are able to suppress the fungal growth and therefore could be used as biocontrol dressings in agriculture.

## 5. Future Outlook

The recent progress in the electrospinning process and development of electrospun polymer materials impeding the growth of pathogenic fungi was summarized in this review. The use of electrospun active dressings in agriculture is a relatively novel approach which seems, however, very promising. Plant protection is the purpose, but this purpose could be achieved with appropriate choice of the polymers and biologically active substances with proper characteristics and activities that are allowed for use in agriculture. Moreover, depending on the composition and purpose, industrial prefabricated dressings or those that are applied directly can be used. For the direct application on plants, a portable electrospinning device needs to be created that can be used in the field to cover the plants or some part of them. These kinds of devices are still at an early stage of development, applied up to now in medicine for the fabrication of personalized wound dressing. However, in the near future, they could become an attractive alternative to classical electrospinning setups because they are small, lightweight, easy to use, flexible to move and carry, and easy to operate. Undoubtedly, the electrospun active dressings created on the basis of biopolymers and biologically active substances by using portable electrospinning apparatuses possess great potential for application in medicine, pharmacy, food-packaging, and agriculture. To enable further development towards the creation of electrospun active dressing for plant protection in real agricultural conditions, further studies in the field are required.

## 6. Conclusions

The present review summarizes the progress in the electrospinning process and discusses the potential applications of the electrospun fibrous materials in agriculture and, in particular, their potential to impede the penetration and growth of pathogenic fungi such as *P. chlamydospora* and *P. aleophilum*. Different kinds of nontoxic and biocompatible polymers and antifungal agents (polyhexamethyleneguanidine; 8-hydroxyquinoline derivatives; nanoparticles of metal oxides) are proposed for the creation of the nonwovens by applying electrospinning or simultaneous electrospinning and electrospraying. Moreover, the successful incorporation of a bioagent, *T. asperellum,* into the fibrous mats, able to suppress the growth of the pathogenic fungi, was achieved. Using the biocontrol agents results in crop increase and diminishes the pollution of the environment with pesticides. The presented results reveal the great potential of the electrospun fibrous materials to serve as active dressings for protection of agricultural crops against the penetration and growth of plant pathogenic fungi.

## Data Availability

Not applicable.
